# Antibacterial Activity of Commercial Dentine Bonding Systems against *E. faecalis*–Flow Cytometry Study

**DOI:** 10.3390/ma10050481

**Published:** 2017-04-29

**Authors:** Monika Lukomska-Szymanska, Magdalena Konieczka, Beata Zarzycka, Barbara Lapinska, Janina Grzegorczyk, Jerzy Sokolowski

**Affiliations:** 1 Department of General Dentistry, Medical University of Lodz, 92-213 Lodz, Poland; barbara.lapinska@umed.lodz.pl (B.L.); jerzy.sokolowski@umed.lodz.pl (J.S.); 2 Department of Microbiology and Laboratory Medical Immunology, Medical University of Lodz, 92-213 Lodz, Poland; magdalena.konieczka@umed.lodz.pl (M.K.); beata.zarzycka@umed.lodz.pl (B.Z.); janina.grzegorczyk@umed.lodz.pl (J.G.)

**Keywords:** dental bonding systems, flow cytometry, *E. faecalis*, antibacterial activity

## Abstract

Literature presents inconsistent results on the antibacterial activity of dentine bonding systems (DBS). Antibacterial activity of adhesive systems depends on several factors, including composition and acidity. Flow cytometry is a novel detection method to measure multiple characteristics of a single cell: total cell number, structural (size, shape), and functional parameters (viability, cell cycle). The LIVE/DEAD^®^ BacLight™ bacterial viability assay was used to evaluate an antibacterial activity of DBS by assessing physical membrane disruption of bacteria mediated by DBS. Ten commercial DBSs: four total-etching (TE), four self-etching (SE) and two selective enamel etching (SEE) were tested. Both total-etching DBS ExciTE F and OptiBond Solo Plus showed comparatively low antibacterial activity against *E. faecalis*. The lowest activity of all tested TE systems showed Te-Econom Bond. Among SE DBS, G-ænial Bond (92.24% dead cells) followed by Clearfil S3 Bond Plus (88.02%) and Panavia F 2.0 ED Primer II (86.67%) showed the highest antibacterial activity against *E. faecalis*, which was comparable to isopropranol (positive control). In the present study, self-etching DBS exhibited higher antimicrobial activity than tested total-etching adhesives against *E. faecalis*.

## 1. Introduction

Clinical success relies on the durable and resistant composite-tooth interface. Dentine bonding systems (DBS) are applied to create hybrid layer, that is responsible for sealing dentine or/and enamel and composite. Residual bacteria left on the cavity surface may cause damage to adhesive interface. Therefore, it is crucial to apply adhesives with good antibacterial properties on the cavity surface in deep cavities.

Literature presents inconsistent results concerning the antibacterial activity of bonding systems [1,2,3,4,5,6,7]. Some adhesives possess antibacterial properties, but some do not exhibit such properties at all. The application of the latter may negatively influence clinical outcomes. Antibacterial activity of DBS depends on several factors, including composition and acidity. Adhesion promoting, acidic monomers, containing phosphoric, carboxylic or acrylic portions in the molecules, are considered to be responsible for the antibacterial effect of the primers or adhesive solutions [8]. Moreover, most DBAs and dental composites have extensive cytotoxic effects on human dental pulp mesenchymal stem cells and even on salivary cells [9,10,11].

Most microbiological studies on antibacterial activity of DBS use agar diffusion test (ADT) [2,5,8,12,13,14,15,16,17,18,19,20,21,22,23,24,25,26,27,28,29,30,31,32,33,34,35]. The method involves agar plates that are inoculated with a standardized inoculum of the test microorganism. Next, filter paper discs containing the tested compound are placed on the agar surface. DBS is dropped with micropipettes on the paper disks or into the wells in the agar surface. While Petri dishes are incubated under suitable conditions, antimicrobial agent diffuses into agar and inhibits germination and growth of the test microorganism. The diameter of inhibition growth zone is measured [36]. This method measures the release of antibacterial substances, but does not indicate whether it has bactericidal or bacteriostatic activity. Moreover, paper disk hampers polymerization and residual monomers may be released inhibiting bacterial growth, although the polymerized DBS exhibits no or weak antibacterial action [37].

A method that allows to differentiate between bactericidal and bacteriostatic effect of tested material is direct contact test (DCT). The direct contact test is based on turbidometric determination of bacterial growth in 96-well microtiter plates. The kinetics of the outgrowth in each well is monitored at 600 nm at 37 °C and recorded every 30 min using a temperature controlled microplate spectrophotometer [38]. It provides a quantitative measure of antibacterial activity of the tested material that remains in direct and close contact with the microorganisms. The method is considered the most valuable in vitro test of the antimicrobial properties of solid dental materials such as restorative materials, endodontic sealers, or cements [38,39,40,41]. Since it is independent of their diffusion properties, may be more suitable for such testing than ADT [41]. Yet, the effect of material components that are capable of diffusing into the liquid medium are also measured in DCT [42].

No correlation between ADT and fluorescence essay was observed [43]. Therefore, the reliability of ADT was questioned and other methods using fluorescence including flow cytometry were recommended [36,43,44,45,46].

Although flow cytometry (FC) has been used in medical diagnostics for many years, is has not been used to estimate microbiological properties of dental materials [47,48,49,50]. The flow cytometer uses lasers and different detectors for light scatter gating and fluorescence detection [51]. Bacterial cells can be counted and characterized (evaluation of structural and functional parameters). Viability is the key cell function investigated in microbiology. The LIVE/DEAD^®^ BacLight™ Bacterial Viability Kit helps to distinguish cells with an intact from those with a compromised membrane. This kit utilizes a mixture of two nucleic acid stains: SYTO9 (green fluorescent dye) and propidium iodide (PI) (red fluorescent dye) for viability determination [52,53]. The SYTO9 can penetrate most cytoplasmic membranes freely, even in cells with membrane integrity, and bind to DNA. The propidium iodide can stain cells with a compromised, permeable membrane only and then stain the nucleic acid. A flow cytometry can identify cell viability by assessing the intensity of the fluorescent staining. The result is that cells with a membrane defect are recognised as dead (non-viable); and cells with an intact membrane as alive (viable) [54].

The aim of the study was to evaluate antibacterial properties of different commercial dentine bonding systems: total-etching (TE), self-etching (SE), and selective enamel etching (SEE), against *E. faecalis* using flow cytometry.

## 2. Results

**Figure 1:**
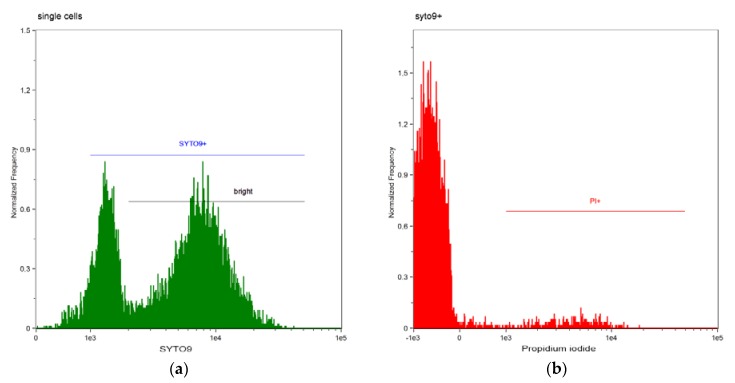
Flow cytometry analysis of *E. faecalis* cell-suspension after 60 min incubation with NaCl (a single experiment example). (**a**) Cells labelled green (SYTO9+); (**b**) Cells labelled red (PI).

**Figure 2:**
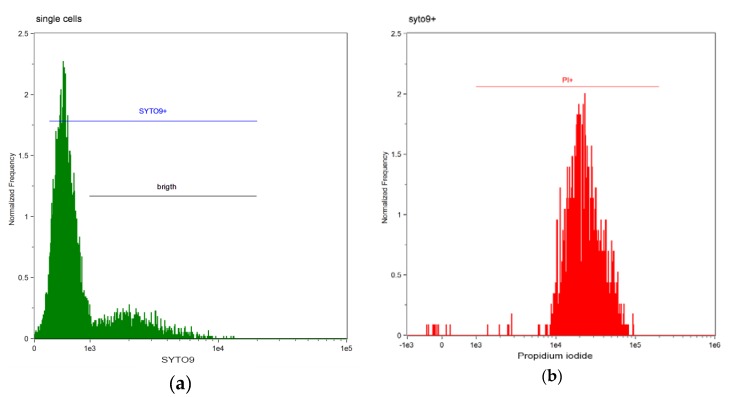
Flow cytometry analysis of *E. faecalis* cell-suspension after 60 min incubation with G-ænial Bond (a single experiment example). (**a**) Cells labelled green (SYTO9+); (**b**) Cells labelled red (PI).

Representative results of flow cytometry analysis for saline and commercial SE dental bonding system (G-ænial Bond) are shown on Figure 1 and Figure 2.

**Figure 3:**
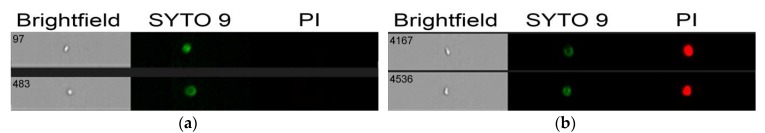
Image gallery of both: (**a**) live, labelled green; (**b**) dead, labelled red and less green cells of *E. faecalis*.

All cells, both living and dead, are green stained by SYTO9 with varying intensity of fluorescence (low and bright) (Figure 1a). Among cells gated as green labelled at the 1a histogram (SYTO9+), regardless of the green staining intensity, dead cells were gated as cells with bright intensity of the red staining (PI) (Figure 1b). Sample analysis after incubation with saline shows very small amount of cells, labelled with red fluorescence (3.82% dead cells), which means that almost all cells were alive in this sample (Figure 3a).

Among cells gated as green labelled (SYTO9+), regardless of the green staining intensity, dead cells were gated as cells with bright intensity of the red staining (PI) and less green fluorescence (Figure 2b and Figure 3b). Sample analysis after incubation with G-ænial Bond shows that almost all cells are dead (98.16% dead cells).

**Figure 4:**
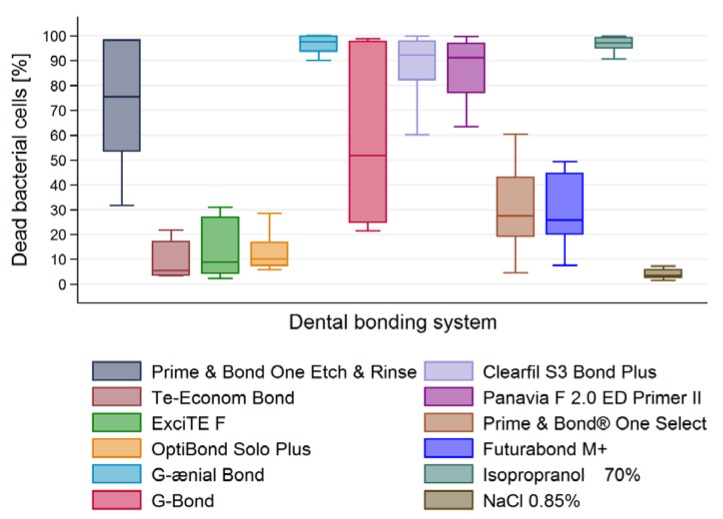
Assessment of dead bacterial cells [%] after incubation with all tested DBS.

Results shown in Figure 1 and Figure 2 are confirmed by the labelling of cells presented in Figure 3. Numerical data of dead bacterial cells [%] resulting from antibacterial activity of DBS were presented in Table 1 and Figure 4.

Among all tested DBS, the highest antibacterial activity against *E. faecalis* was observed for G-ænial Bond (92.24% dead cells) and it was comparable to 70% isopropranol activity (positive control) (Table 1, Figure 4). Both self-etching DBSs, Clearfil S3 Bond Plus (88.02%) and Panavia F 2.0 ED Primer II (86.67%) followed by Prime&Bond one Etch&Rinse (72.04%) exhibited comparatively high antibacterial activity. The lowest activity was observed for Te-Econom Bond (9.19%); it was almost as low as for saline. 

For the general model used (Kruskal-Wallis test) the differences in antibacterial activity of tested DBS were considered statistically significant (*p* < 0.001). In order to pinpoint meaningful pairwise differences, Fisher’s protected least-significant differences (LSD) were computed (with a cut-off value for statistical significance set at *p* < 0.05) (Table 2).

**Figure 5:**
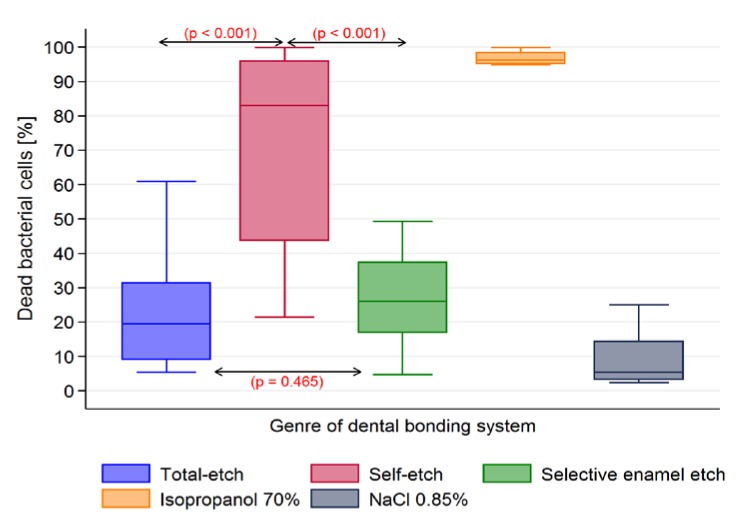
Assessment of dead bacterial cells [%] after incubation–group comparison of types (TE, SE, SEE) of tested DBS.

In general, self-etching DBS exhibited higher antimicrobial activity than other tested adhesives (*p* < 0.001) (Figure 5).

## 3. Discussion

*Enterococcus faecalis* is one of the crucial pathogens present in the oral cavity [55,56]. It is suggested that *E. faecalis* plays an important role in the etiology of post-treatment apical periodontitis since it prevails in secondary endodontic infections rather than in primary infections. *E. faecalis* may enter the root-filled canal via coronal leakage during or after root-canal treatment as secondary invaders [57]. Since *E. faecalis* poses intrinsic and acquired resistance to many antibiotics and other antimicrobial substances including chloramphenicol, tetracyclines, macrolides, and clindamycin [56,58], bacteria is difficult to eradicate. Additionally, some of the irrigants used in root canal treatment exhibited little or no bactericidal effect against *E. faecalis* [59]. The study on bactericidal activity of irrigants, using the dilution-neutralization method, showed that 17% EDTA even after 60-min incubation had no bactericidal effect, while 25% citric acid solution and 10% citric acid solution after 3 and 10 min respectively showed bactericidal activity against *E. faecalis*. Similar activity was found for 2.5% and 5.0% phosphoric acid after 5 and 3 min, respectively [59]. Other irrigants, like 5.25% NaOCl, were proven to be effective after 2 min contact with bacteria [60]. Gomes et al. [61], testing irrigants in cell suspension of *E. faecalis*, reported that both NaOCl and CHX were effective in *E. faecalis* eradication. Furthermore, Vahdaty et al. [62], using infected tooth model, tested that either CHX or NaOCl (at similar concentrations of 0.2% and 2%) were equally effective against *E. faecalis*, but the reduction of bacteria counts still left up to 50% of the dentine infected. Noites et al. [63] confirmed these findings, also using the infected tooth model, reporting that 2% CHX as well as NaOCl (1–5%) were almost ineffective for *E. faecalis*. The same researchers, who used flow cytometry, proved the effectiveness of 2% CHX irrigation followed by gaseous ozone application on *E. faecalis* complete elimination from root canals [63]. Popular dressings used during endodontic treatment, such as calcium hydroxide mixed with distilled water or with 0.2% chlorhexidine, failed to eliminate *E. faecalis* in disinfected dentinal tubules [64,65]. Other results showed that camphorated paramonochlorophenol increased the antibacterial effects of calcium hydroxide against *E. faecalis* [66].

Nowadays, minimally invasive dentistry as well as the control of bacterial infection of dentine impose the urge to develop bonding systems possessing antibacterial properties. In order to achieve the goal, incorporation of antibacterial component in DBS composition was performed [1,25,31,42,67,68,69,70], involving the use of monomers like methacryloxylethyl cetyl dimethyl ammonium chloride (DMAE-CB) or methacyloyloxdodecyl pyridinium bromide (MDPB) that were immobilized in the primer, or fluoride. MDPB is an antibacterial monomer that is considered to possess significant bactericidal activity against crucial pathogens present in oral cavity (*S. mutans, L. casei, L. acidophilus, E. faecalis*) [25,42,67,71,72,73,74]. While, DMAE-CB is a monomer that contains quaternary ammonium, which exhibits antibacterial activity [75]. Therefore, bonding systems with good antibacterial properties against oral pathogens are crucial.

Although the literature on antibacterial properties of DBS is abundant, it is inhomogeneous and sometimes difficult to interpret [76]. Researchers used different study methods, bacterial strains, bonding systems, times, and conditions of incubation. Moreover, progress in scientific analysis of materials and increasing demands of the dental market result in manufacturing new or upgraded products that should meet customer demands.

Agar diffusion test (ADT) is a simple, qualitative, economic and the most commonly used method assessing antibacterial properties of DBS. Other quantitative methods include broth culture test, spectrophotometery, determining: colony forming units, maximum or minimum inhibitory concentration (MIC), as well as minimum bactericidal concentration, direct contact test (DCT) or SEM [76]. Moreover, some studies [29,31,71,77] used Live/Dead BacLight^®^ bacterial viability stain obtaining the amount of viable bacteria by measuring fluorescence on a fluorometer [29], examining bacteria cell with an epifluorescence microscope [77], or visualizing bacterial biofilm by confocal laser scanning microscopy (CLSM) [31,71].

Flow cytometry is a novel detection method to measure multiple characteristics of a single cell: total cell number, structural (size, shape), and functional parameters (viability, cell cycle). On one hand, FC is a high speed analysis providing results that can be clearly interpreted. On the other hand, it demands very expensive and sophisticated instruments and a highly trained specialist. Nowadays, it is applied in diagnostics and many areas of science such as haematology, transplantology, immunology, or microbiology. It is a very helpful diagnostic method to evaluate human blood cells, especially immunocompetent, even after treating with various agents like microbial cells are tested [78,79,80,81,82]. However, dental materials have not been investigated using this technology. Therefore, application of flow cytometry study in the antibacterial evaluation of DBS may be a promising microbiological method.

Publications on antibacterial activity of commercial bonding systems against *E. faecalis* are limited. Syntac Adhesive, fourth generation total-etching DBS, was found to exhibit good antibacterial properties against *E. faecalis* and disinfect dentine blocks [83]. The effect can be explained by the content of glutaraldehyde, that has high antibacterial efficacy even at low concentrations. In the present study, Prime&Bond one Etch&Rinse showed the highest antibacterial activity against *E. faecalis*, among total-etching DBS. Vaidyanathan et al. [32] observed that the majority of the TE DBS tested exhibited antimicrobial activity in the in vitro models (DCT and ADT), while in ex vivo model they exhibited the level of activity comparable to the etchant (37.5% phosphoric acid). Only OptiBond Solo Plus exhibited antimicrobial activity in both in vitro (DCT, ADT) and in ex vivo assays, having a stronger effect than the etchant alone. The authors argued that using the ex vivo model provides more accurate determination of DBS’ antibacterial activity. However, both total-etching adhesives, Prime&Bond NT and OptiBond Solo Plus exhibited low antibacterial properties [32]. Disk Diffusion Method revealed strong antibacterial properties (against among others *S. mutans, L. acidophilus*) for ExciTE [84,85], which was not observed by other researchers [5,28,32,86]. In the present study, both ExciTE F and OptiBond Solo Plus showed comparatively low antibacterial activity against *E. faecalis*. The lowest activity of all tested total-etching systems showed Te-Econom Bond. Baca et al. [87], using MBEC™ High-throughput (HTP) assay, observed the smallest amount of *E. faecalis* biofilm was formed on Clearfil Protect Bond and ExciTE, while the greatest biofilm amount was formed on Futurabond.

Antibacterial activity of DBS depends on several factors, including composition and acidity [2]. While the content of acidic primer in self-etching DBS causes demineralization of the smear layer and the dentine, allowing for simultaneous etching and priming, non-rinsing procedure may result in bacteria retention at the tooth-restoration interface. Most of the studies indicate that low pH of the primers is the main factor in bacteria growth inhibition [6,16,19,73,85].

All tested commercial self-etching DBSs were ‘mild’ self-etch systems, having a pH of around 2 [88]. It is worth emphasizing, that the self-etching DBSs exhibited in general significantly higher antimicrobial activity against *E. faecalis* than tested total-etching adhesives (*p* < 0.001). Self-etching adhesives like G-Bond, G-ænial Bond, Adper Easy One, Xeno V, Clearfil S3 Bond exhibited low [2,6,89] or no antibacterial activity against *S. mutans* [4,6,67,90]. These adhesives showed immediate bactericidal effect on *S. mutans*, in ADT and DCT, that only lasts up to 24–48 h [2,7]. It is assumed that the antibacterial component might decompose with time into surrounding media at different rates [2]. In the present study, using flow cytometry, G-ænial Bond followed by Clearfil S3 Bond Plus and Panavia F 2.0 ED Primer II showed the highest antibacterial activity against *E. faecalis*, that was comparable to isopropranol (positive control). Clearfil Protect Bond and Clearfil SE Bond exhibited comparable antibacterial properties against *E. faecalis* in DCT, but no action was found in ADT [42]. Another study showed that SE bonding systems (Clearfil SE Bond and Clearfil Protect Bond) did not inhibit caries caused by *S. mutans*, even though MDPB- and F-containing DBS (Clearfil Protect Bond), decreased glucan synthesis [24]. Similar results were confirmed by other authors [15,91]. Carvalho et al. [71] who used confocal laser scanning microscopy found that even though viable bacteria were present 20 s after application of Clearfil SE Bond on dentine, their count did not increase during next 10 min. The application of Clearfil Protect Bond (containing MDPB) resulted in gradual increase of non-viable bacteria over 10 min.

Both commercial DBS tested in the present study (Prime&Bond One Select and Futurabond M+), that can be used in all etching techniques, had low antibacterial activity against *E. faecalis*. Futurabond is claimed to have comparable antibacterial activity with 0.012% CHX when tested with ADT [6] and proved to have the best inhibitory properties against *S. mutans* in DCT [32].

## 4. Materials and Methods 

### 4.1. Eluate Preparation

The dental bonding systems used in the study are presented in the Table 3.

Each DBS was loaded in 50 µL measures into round-shaped tubes and distributed evenly. After polymerization according to manufacturer’s instructions (20 s or 30 s) 2 mL sterile buffered saline (OXOID, Basingstoke, GB) were aliquoted and incubated for 24 h in 35 °C. The next day, samples were centrifuged (2000 rpm, 5 min) to obtain eluates utilized in further experiments.

### 4.2. Microbank System

Microbiological studies were conducted on reference strain *Enterococcus faecalis* ATCC 29212. The strain was stored in Microbank system (Biocorp, Warsaw, Poland) as described by Łukomska-Szymańska et al. [96]. Vials with the bacteria strain in cryopreservation media were stored in freezer at −80 °C.

### 4.3. Bacteria Suspension Preparation

The bacteria strain of *E. faecalis* from Microbank system was revived on culture medium, Columbia agar (Becton Dickinson, Becton Dickinson, Franklin Lakes, NJ, USA) in aerobic conditions in 35 °C. After first 18-h cultivation, next 18-h bacterial culture was done at the new medium plate to obtain reproducibility of the method. Each experiment was performed from the same, second recultivation. The bacterial emulsion harvested from the medium was used to gain suspension in McFarland standard 0.5 in sterile buffered saline. That bacteria suspension was tested with 10 DBS (Table 1).

### 4.4. Bacteria Incubation

Bacterial suspension measures of 1 mL were aliquoted into 12 sterile tubes and centrifuged at 10,000 *g* for 2 min. The supernatants were discarded and then 1 mL: 0.85% NaCl, 70% isopropanol or the eluate prepared from bonding system was added respectively to resuspend the pellets. Well mixed samples were incubated for 1 h in 35 °C, mixing every 15 min. Next, both controls (negative-only with saline; positive-with isopropanol) and test samples were centrifuged (10,000 *g*, 2 min) and washed with PBS without Ca and Mg ions (PAN Biotech, Aidenbach, Germany). After the washing step, 300 µL PBS was added to the pellet of bacteria. All samples were analyzed with LIVE/DEAD flow cytometry method.

### 4.5. Flow Cytometry Staining Procedure

Following the manufacturer’s instructions, a LIVE/DEAD^®^ BacLight™ Bacterial Viability Kit (Molecular Probes, Life Technologies, Eugene, OR, USA) was used for the analysis. Bacteria suspension (150 µL) was stained with 5 µL SYTO9 and propidium iodide (PI) and incubated for 15 min in the dark at room temperature. Flow cytometric measurements were performed on a ImageStreamX Mark II (ISX-MkII) (Amnis, EMD Millipore, Seattle, WA, USA) with 488 nm excitation from a blue laser, at 50 mW, counting 10,000 objects. The fluorescence was collected in the green and red channels. The bacterial cells with intact cell membrane show bright green fluorescence (live cells), whereas the bacteria with damaged cytoplasmic membranes exhibit much less green fluorescence and bright red fluorescence (dead cells). The results were expressed as the percentage of dead bacterial cells and were analyzed using IDEAS^®^ 6.1 (Image Data Exploration and Analysis Software). All experiments were performed in duplicate. For the single-color histogram charts, a representative experiment is shown. The numeric results including standard deviation are listed as a mean.

### 4.6. Statistical Analysis

The growth inhibition zone was measured in millimeters in two perpendicular lines intersecting in the middle of the investigated zone. The dental bonding systems employed in the study were codified as a discrete variable. Due to small sample sizes and eventually an abnormal distribution of the numerical data, non-parametric tests were performed. In the case of comparisons with two independent groups (two bonding systems), the Mann–Whitney–Wilcoxon rank-sum test was fitted. When dealing with three or more independent variables (three or more bonding systems), the Kruskal–Wallis rank test was performed. In both cases, in order to enhance the statistical power of the computations and diminish a faulty inference, the outcome was obtained through bootstrapping.

A level of *p* < 0.05 was considered statistically significant. All the statistical procedures were carried out using Stata^®^/Special Edition, release 14.2 (StataCorp LP, College Station, TX, USA).

## 5. Conclusions

(1) Flow cytometry seemed to be a very useful evaluation method of antibacterial activity of dentine bonding systems.
(2) Self-etching bonding systems exhibit significantly higher antibacterial activity against *E. faecalis* in comparison to total-etching DBS.
(3) The highest percentage of dead bacteria cells was found for G-ænial Bond, while the lowest–for Te-Econom Bond.

## Figures and Tables

**Table 1:** Antibacterial activity of DBS against *E. faecalis*-Statistical parameters.

DBS		Dead Bacterial Cells (%)			
		M	SD	CV	Min.–Max.
TE	Prime&Bond one Etch&Rinse	72.04	27.47	38.13%	31.80–98.20
	Te-Econom Bond	9.19	7.31	79.53%	3.43–21.71
	ExciTE F	13.76	12.26	89.11%	2.29–30.98
	OptiBond Solo Plus	13.13	8.39	63.91%	5.88–28.45
SE	G-ænial Bond	92.24	3.64	3.78%	90.00–99.92
	G-Bond	60.46	35.16	58.16%	21.38–98.74
	Clearfil S3 Bond Plus	88.02	13.70	15.57%	60.20–99.82
	Panavia F 2.0 ED Primer II	86.67	14.20	16.39%	63.48–99.73
SEE	Prime&Bond^®^ One Select	30.53	18.86	61.78%	4.63–60.23
	Futurabond M+	28.87	15.58	53.96%	7.57–49.23
Control	Isopropranol 70%	95.41	5.96	6.24%	76.75–99.81
	NaCl 0.85%	6.56	7.25	110.44%	1.49–25.00

M–mean; SD–standard deviation; CV–coefficient of variation.

**Table 2:** Levels of statistical significance for post hoc pairwise comparisons of percentages of dead cells, based on Fisher’s protected least-significant difference (LSD).

DBS	Prime&Bond one Etch&Rinse	Te-Econom Bond	ExciTE F	OptiBond Solo Plus	G-ænial Bond	G-Bond	Clearfil S3 Bond Plus	Panavia F 2.0 ED Primer II	Prime&Bond^®^ One Select	Futura Bond M+	Isopropanol 70%	NaCl 0.85%
Prime&Bond one Etch&Rinse	-	**=0.006**	**=0.023**	**=0.010**	**=0.002**	**=0.010**	=0.138	=0.112	**<0.001**	**=0.016**	**=0.001**	**<0.001**
Te-Econom Bond	**<0.001**	-	=0.575	=0.844	**<0.001**	=0.056	**<0.001**	**<0.001**	=0.617	**=0.038**	**<0.001**	=0.657
ExciTE F	**<0.001**	=0.575	-	=0.715	**<0.001**	=0.170	**<0.001**	**<0.001**	=0.952	=0.122	**<0.001**	=0.278
OptiBond Solo Plus	**<0.001**	=0.844	=0.715	-	**<0.001**	0.085	**<0.001**	**<0.001**	=0.761	=0.058	**<0.001**	=0.503
G-ænial Bond	**=0.002**	**<0.001**	**=0.002**	**<0.001**	-	**=0.020**	=0.077	=0.096	**=0.001**	**=0.023**	=0.921	**<0.001**
G-Bond	**=0.010**	=0.056	=0.170	=0.085	**<0.001**	-	**<0.001**	**<0.001**	=0.153	=0.857	**<0.001**	**=0.010**
Clearfil S3 Bond Plus	=0.138	**=0.001**	**=0.006**	**=0.002**	=0.077	**<0.001**	-	=0.913	**=0.004**	**<0.001**	=0.052	**<0.001**
Panavia F 2.0 ED Primer II	=0.112	**<0.001**	**=0.008**	**=0.003**	=0.096	**<0.001**	=0.913	-	**<0.001**	**<0.001**	=0.068	**<0.001**
Prime&Bond^®^ One Select	**<0.001**	=0.617	=0.952	=0.761	**<0.001**	=0.153	**<0.001**	**<0.001**	-	=0.109	**<0.001**	=0.309
Futura bond M+	**=0.016**	**=0.038**	=0.122	=0.058	**<0.001**	=0.857	**<0.001**	**<0.001**	=0.109	-	**<0.001**	**=0.006**
Isopropanol 70%	**=0.001**	**<0.001**	**<0.001**	**<0.001**	=0.921	**<0.001**	=0.052	=0.068	**<0.001**	**<0.001**	-	**<0.001**
NaCl 0.85%	**<0.001**	=0.657	=0.278	=0.503	**<0.001**	**=0.010**	**<0.001**	**<0.001**	=0.309	**=0.006**	**<0.001**	-

**Table 3:** Commercial bonding systems used in the study.

Name	Manufacturer	Number of Components	Type	Resin/Monomer	pH	Mode of Etching		
						Total-Etching	Self-Etching	Selective Enamel Etching
Prime&Bond One Etch&Rinse	Dentsply, UK	1	2-step	TCB resin, phosphoric acid modified acrylate resin (PENTA), UDMA, TEGDMA, HEMA	2.5 *	+		
Te-Econom Bond	Ivoclar Vivadent, Germany	1	2-step	HEMA, di- and mono-methacrylates	2.6 *	+		
ExciTE^®^ F	Ivoclar Vivadent, Germany	1	2-step	Bis-GMA, HEMA, phosphoric acid acrylate, dimethacrylates	2.5 *	+		
OptiBond™ Solo Plus	Kerr/USA	1	2-step	Bis-GMA, GPDM, HEMA	2.2 *	+		
G-ænial^®^ Bond	GC, Japan	1	1-step	4-MET, phosphoric acid ester monomer	1.5 [92]		+	
G-Bond^®^	GC, Japan	1	1-step	UDMA	2.0 [93]		+	
Clearfil S3 Bond Plus	Kuraray America, USA	1	1-step	MDP, Bis-GMA, HEMA	2.3 *		+	
Panavia F 2.0 ED Primer II	Kuraray America, USA	2 (A + B)	2-step	2-Hydroxyethyl methacrylate, 10-methacryloyloxydecyl dihydrogen phosphate *N*-Methacryloyl-5-aminosalicylic acid	2.4 [94]		+	
Prime&Bond^®^ One Select	Dentsply, UK	1	1- or 2-step	bifunctional acrylate, acidic acrylate, phosphoric acid ester	1.6 *	+	+	+
Futurabond M+	VOCO, Germany	1	1- or 2-step	Bis-GMA, HEMA	2.0 [95]	+	+	+

* Information obtained from the manufacturer (safety data sheet).
